# Cutaneous Microbiome Profiles Following Chlorhexidine Treatment in a 72-Hour Daily Follow-Up Paired Design: a Pilot Study

**DOI:** 10.1128/spectrum.01753-21

**Published:** 2022-06-21

**Authors:** Jean-Luc C. Mougeot, Micaela F. Beckman, Farah Bahrani Mougeot, James M. Horton

**Affiliations:** a Carolinas Medical Center, Atrium Health, Charlotte, North Carolina, USA; University of Illinois at Urbana Champaign

**Keywords:** bloodstream infections, chlorhexidine, skin microbiome, 16S rRNA gene sequencing

## Abstract

Venous catheter-related bloodstream infections represent a significant problem in the United States. Our objective was to determine daily changes in skin microbiome profiles up to 72h postchlorhexidine treatment. Left and right forearm skin swab samples were obtained from 10 healthy volunteers over 72h at 24h intervals. Dorsal surface of left arm was treated with chlorohexidine gluconate (CHG) at initial time point (T = 0), while the right arm remained untreated (control). Swab samples were obtained shortly before (T = 0) and after CHG treatment (T = 24–48-72h). Bacterial DNA extraction, 16S rRNA gene V1-V3 sequencing and taxonomic annotation were performed using ZymoBIOMICS pipeline. PERMANOVA, linear discriminant and bacterial interaction network analyses were performed. A total of 13 total phyla, 273 genera, and 950 total species were detected across all time points, CHG-treated or CHG-untreated. Most abundant species included Cutibacterium acnes, Staphylococcus epidermidis, and Rothia Mucilaginosa. Low biomass-related inconsistent taxa detection was observed. PERMANOVA suggested a marginal difference between CHG-treated and CHG-untreated microbiome profiles (Genera: P(perm) = 0.0531; Species: P(perm) = 0.0450). Bacterial interaction network guided PERMANOVA analyses detected a microbiome change over time, suggesting a consistent CHG treatment-specific change. LEfSe identified Finegoldia magna, Bacillus pumilus, Bacillus thermoamylovorans as the only distinctive species. These species were more abundant and/or present post-CHG treatment in the CHG-treated group. These findings suggest that the skin microbiome was not significantly different 24, 48, or 72h after CHG treatment. Previous culture-based studies have found similar results after 24h. Future studies will be needed to determine the mechanisms of bacterial regrowth after CHG treatment.

**IMPORTANCE** Annually, over 80,000 central line infections occur in the United States. Understanding the pathogenesis of these infections is crucial. Chlorhexidine is the most commonly used skin preparation before line placement. We hypothesized that the use of chlorhexidine and dressings will alter the normal arm skin microbiome over a period of 72h. We used 16S-rRNA gene next generation sequencing (NGS) to determine the forearm skin microbiome of volunteers. The left arm was swabbed with chlorhexidine and the right arm served as control. The skin microbiome returned to normal after 24h. Our NGS results confirm findings of two previous culture-based studies. Relative abundance of *Bacillus* spp. in the chlorhexidine-treated samples was increased, consistent with one previous study. Based on the results of this pilot study, we will need to measure viable bacteria during a 24h time course following chlorhexidine treatment to understand the source of skin microbiome replenishment.

## INTRODUCTION

Annually about 80,000 central venous catheter-related bloodstream infections occur in the United States (1). Bloodstream infection from central venous catheters (CVCs) comes from four sources: colonization from the skin, the catheter hub contamination, hematogenous infections from another skin site, and, rarely, contamination of the infusate (2). The most common source of central line related infections is colonization of the catheter by microorganisms from patient’s skin. Several studies have found a strong correlation between skin colonization and both catheter colonization and subsequent catheter-related infection, especially with short-term intravascular devices (3–5).

From the moment of birth, our bodies are colonized with bacteria on the skin and in the intestines. These normal skin bacteria are very complex. The 16S rRNA gene sequencing analysis of superficial skin bacteria, has identified approximately 182 different species in left and right forearms of healthy subjects (6). These bacterial species are important as they provide defense against more harmful species such as MRSA.

Wound dressings were invented in the time of the ancient Sumerians, and the sterile dressing was developed in the late 1800s to keep harmful bacteria out of wounds (6–11). Several studies (12–14) used cultures to analyze skin microbes after CHG and found resilience of the microbiome. Those studies have some limits because patients were on systemic antibiotics, or they detected a limited number of bacterial species.

We hypothesized that use of chlorhexidine and dressings will alter the normal arm skin microbiome as measured by 16S rRNA gene sequencing with detectable changes over a period of 72 h. V1-V3 16S rRNA bacterial gene next generation sequencing was performed using Illumina MiSeq platform.

## RESULTS

### Detection of species and genera across time points.

Demographics and clinical characteristics of our healthy volunteers’ cohort (N = 10) are presented in Table 1. A total of 15 phyla (13 CHG-Tr, 15 CHG-Un, 10 in common), 373 genera (287 CHG-Tr, 239 CHG-Un, 153 in common), and 950 taxa (643 CHG-Tr, 534 CHG-Un, 227 in common), were identified in all healthy volunteers (HVs, N = 10) across all four time points combined. The most abundant species-level taxa was Cutibacterium acnes followed by Staphylococcus epidermis, as previously reported in skin microbiome studies (15, 16). The top 50 most abundant species included Rothia mucilaginosa also previously found among the most abundant species in previous studies (15, 16), and Corynebacterium mucifaciens, Staphylococcus hominis, Streptococcus mitis, which to our knowledge have not been identified in the forearms of HVs using 16S rRNA gene metagenomic sequencing.

**TABLE 1:** Demographics of healthy volunteers recruited for skin microbiome analysis^*a*^

Variable	CHG-treated^*b*^		CHG-untreated^*c*^
No. of samples	4 × 10		4 × 10
Age [SD]	44.5 [10.87]		44.5 [10.87]
Gender (M/F)	6/4		6/4

a Age is depicted as an average. M is male, F is female, SD is standard deviation. Healthy volunteers’ demographics (N = 10) and clinical characteristics used for analysis for each group. Samples were collected at four time points T0, T24, T48, and T72.

b CHG-treated represents the Chlorhexidine gluconate (CHG) treatment group.

c CHG-untreated corresponds to the untreated group.

Following a preliminary analysis at the phylum level, HV-205 data were excluded from the analysis due to low level of taxa detection. This resulted in a cohort of 9 HVs for analysis (Supplemental File 1). Indeed, only Actinobacteria, the most predominant phylum, was detected in all eight instances except two in which the second most predominant phylum Firmicutes was detected. All other HVs had at least two phyla detected in both arms for all time points. In addition, no data were obtained for HV-210 for the CHG-untreated group at time point T2, resulting in a data set of 35 samples per arm instead of the 36 possible for the 9 HVs cohort. Furthermore, a data set was generated to include species/genera with more consistent representation across time points based on the criteria that taxa needed to be present in at least 4 of 35 samples in both arms (>10%) (Table 2).

**TABLE 2:** Species and genera detected across time points in Left (CHG-treated) and Right (CHG-untreated) arms swab samples

Time point CHG-Tr & CHG-Un	Total no. detected^*a*^	No. detected in >10% HVs^*b*^	Avg no./HV detected in >10%^*c*^
Species			
CHG-Tr0	229	46	12
CHG-Un0	195	45	11.11
CHG-Tr1	231	46	11.33
CHG-Un1	250	48	13.78
CHG-Tr2	187	46	10.11
CHG-Un2	158	45	14.4
CHG-Tr3	273	49	13.89
CHG-Un3	203	42	11.22
CHG-Tr_cum_^*d*^	465	52	13.77
CHG-Un_cum_^*d*^	534	52	13.63
Genera			
CHG-Tr0	119	37	11.67
CHG-Un0	110	40	12.44
CHG-Tr1	128	41	12.56
CHG-Un1	134	40	12.44
CHG-Tr2	114	36	11
CHG-Un2	81	36	9.67
CHG-Tr3	139	39	12.33
CHG-Un3	111	31	10.56
CHG-Tr_cum_^*d*^	281	41	13.29
CHG-Un_cum_^*d*^	239	41	12.83

a Total numbers of species and genera detected, in each of the four time points, in each arm/group (Left/CHG-treated, CHG-Tr0, CHG-Tr1, CHG-Tr2, CHG-Tr3, and CHG-Tr4; Right/CHG-Untreated, CHG-Un0, CHG-Un1, CHG-Un2, CHG-Un3, and CHG-Un4).

b Species or genera detected in at least 10% of samples (i.e., 4 of 35 samples) across all time points and in common between both groups. HV, healthy volunteer.

c Average numbers of species/genera per subject detected in >10% samples across all time points and in common between CHG-Treated and CHG-Untreated groups.

d Cumulative (cum) numbers of species or genera detected per CHG-treated or CHG-Untreated group.

### Differences in *beta*-diversity.

**FIG 1:**
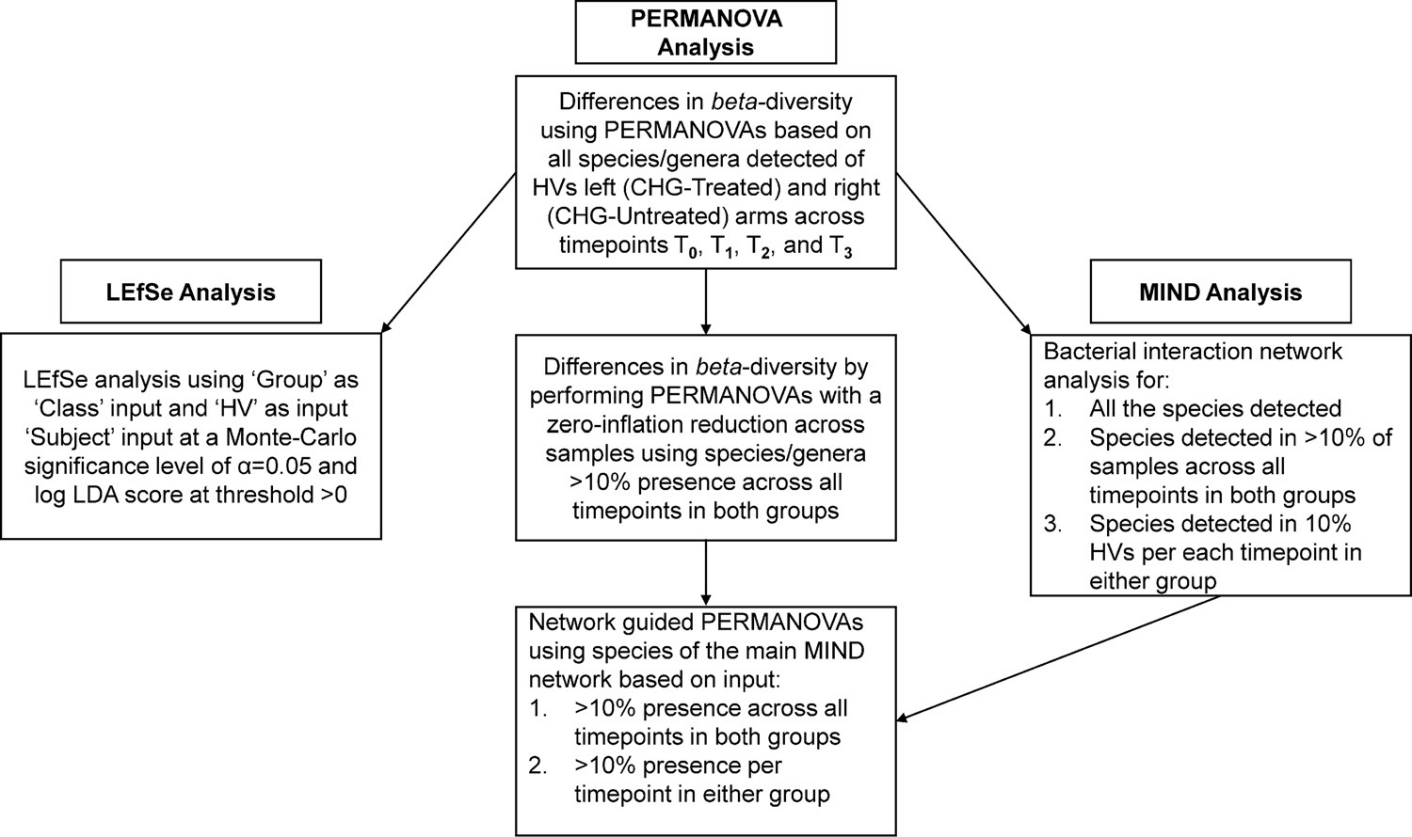
Analytical design of multivariate analysis of chlorhexidine effect on skin microbiome. Multivariate analyses were performed for (i) all species/genera detected in left arm (chlorhexidine treated) of all nine Healthy Volunteers (HVs), (ii) species detected in at least 10% of samples across all time points in both arms, i.e., 4 of 35 samples per each arm, (iii) species detected in at least 10% of HVs per either time point, i.e., 1 of 9 HVs in either arm. Groups were Chlorhexidine (CHG) treated and untreated; MIND_v1.01_ program is Microbial Interaction Network Database (http://www.microbialnet.org/mind.html); LEfSe stands for Linear discriminant analysis (LDA) effect size.

**(i) Conventional PERMANOVA**. Following the preliminary analysis of relative abundance, data from HV-205 and paired data (CHG-Tr2-210 and CHG-Un2-210) were excluded in the PERMANOVA design (i.e., only T0, T1 and T3 data were used). This resulted in the inclusion of CHG-treated and CHG-untreated data for 35 samples each, instead of the 9 × 4 = 36 possible. Our overall PERMANOVA analytical strategy is described in Fig. 1.

PERMANOVA was performed for a series of longitudinal comparisons at the species and genus levels, as shown in Table 3. When using all the species or genera detected across the four time points in PERMANOVA, all comparisons were far from reaching statistical significance, apart from the CHG-TrUn0 versus CHG-TrUn1,2,3 comparison which was marginally significant (Species: P(perm)=0.0450; Genera: P(perm)=0.0531; nonsignificant P(MC)) (Table 3). The results suggest that a very small difference exists between treated and untreated microbiomes overall, but that no change between the time points distinguishing CHG-treated from CHG-untreated could be detected. Additionally, the comparisons CHG-TrUn0 *versus* average of CHG-TrUn1, 2, and 3 data at species and genus levels, did not reach statistical significance either.

**TABLE 3:** PERMANOVA results, species, or genera for all or >10% of CHG-Treated and CHG-Untreated samples^*a*^

Comparison	Group P(Perm)	
	All species^*b*^	All genera^*b*^
CHG-TrUn0 *versus* CHG-TrUn1,2,3	>0.0450	>0.0531
CHG-TrUn0 *versus* CHG-TrUn_avg_	0.3206	0.2976
CHG-TrUn0 *versus* CHG-TrUn1	0.5217	0.5472
CHG-TrUn0 *versus* CHG-TrUn3	0.3440	0.5112
CHG-TrUn0 *versus* CHG-TrUn1,2	0.2694	0.2645
CHG-TrUn0 versus CHG-TrUn1,3	0.0890	0.9799
CHG-TrUn0 *versus* CHG-TrUn2,3	0.2382	0.1887

**Comparison**	**Species CHG-Tr&CHG-U n>10%**	**Genera CHG-Tr&CHG-U n>10%**

CHG-TrUn0 *versus* CHG-TrUn1,2,3	0.4316	0.6475
CHG-TrUn0 *versus* CHG-TrUn_avg_	0.4692	0.5330

a A three-factor fixed design consisting of “Group” (CHG-Treated *versus* CHG-Untreated), “Time point (T**_0_**, T**_1_**, T**_2_**, and T**_3_**), and “Subject” (paired left (CHG-Treated) and right (CHG-Untreated) forearms; N = 9 healthy volunteers) was used as PERMANOVA design. Bray Curtis similarity matrices were derived from square root-transformed relative abundance data. PERMANOVAs were implemented using a mixed-model design, unrestricted permutation of raw data, 9,999 permutations, and a type III partial sum of squares.

b The following longitudinal comparisons were performed: (i) all species (n = 950) or genera (n = 373) detected in all Left (CHG-Tr; CHG-treated) and Right (CHG-Un; CHG-Untreated) arms of nine healthy volunteers; (ii) species (n = 52) or genera (n = 41) present in >10% samples across all time points and in common between both arms. Paired pretreatment data (CHG-TrUn0) were compared to paired CHG-TrUn posttreatment data of one, two or three time points combined, or to the average (avg) relative abundance data derived from the three posttreatment time points. The analysis CHG-TrUn0 *versus* CHG-TrUn2 was excluded due to missing T**_2_** CHG-Un2 data.

In the next level of analysis, to reduce zero-inflation across all samples and increase power by improving the signal to noise ratio, only genera/species with at least 10% presence in all samples (4 of 35 samples CHG-treated and CHG-untreated) were used. Of 373 genera, 61 genera, including 20 that were unique to the treated arm and 49 genera, including 8 that were unique to the CHG-untreated arm (i.e., n = 41 in common), had 10% presence across both CHG-treated and CHG-untreated samples. Of 950 species, 72 species, including 20 unique to the CHG-treated arm and 70 species, including 18 unique to the CHG-untreated arm (i.e., n = 52 in common) had 10% presence across both CHG-treated and CHG-untreated samples. Using common genera (n = 41) or species (n = 52), i.e., including taxa that were more consistently present in the PERMANOVA comparisons CHG-TrUn0 versus CHG-TrUn1, 2, 3 and CHG-TrUn0 to the average of CHG-TrUn1, 2, and 3, did not yield any significant result (Table 3). In addition, by, including in these comparisons species/genera that were unique to either CHG-treated or CHG-untreated arm, as described above, no statistical significance was reached either (data not shown). Overall, the results suggest that no chlorhexidine treatment effect could be detected for this study’s time series, based on described PERMANOVA analyses of the abundance data. Moreover, using Micropower R package, we were able to determine that for 40 samples in each group (CHG-treated and CHG-untreated; 80 samples total), considering the average depth of raw reads or chimera-free sequences identified in the DADA2 inferred sequences, (Table S2), we had 80% power to detect an effect size of 0.003 and 0.009, respectively.

**(ii) Bacterial interaction network guided PERMANOVA**. A microbial interaction network (MIND) guided PERMANOVA analysis was performed with the assumption that more consistency of the skin microbiome and possible treatment-induced changes might be observed at the network level. The online MIND program used processes a list of taxa detected and does not incorporate abundance data in the analysis. Presumably, a network disturbance that would be caused by chlorhexidine treatment across time points might be more detectable or meaningful, by reducing, in the overall analysis, the inconsistent and stochastic species detection/identification. Inconsistent species detection/identification could be due to low microbiome biomass sampling which is characteristic of skin microbiome studies, and the influence of external environmental factors such as exposure to air after showering for example. Fig. S1 presents MINDs of CHG-treated and CHG-untreated arms obtained over time, (i) for the species present in >10% of samples per time point, i.e., in at least one out of nine HVs in either arm (Fig. S2a) and (ii) for the species present in >10% across all time points in both CHG-treated and CHG-untreated arms, i.e., 4 of 35 samples for each arm (Fig. S2b).

In the first (i) comparison (Fig. S2a), CHG-treated MINDs have similar appearance across the posttreatment time points, which differs from CHG-untreated MINDs’ appearance, suggesting a treatment effect. Such presumed effect was not confirmed by PERMANOVA performed on the relative abundance data of 173 species representing the main network nodes, including 72 in common across all time across both arms. The 173 species were presumed to be more consistently present or possibly be more consistently affected by treatment across time points (Table 4).

**TABLE 4:** Species level network guided PERMANOVA results^*a*^

Main network comparison	Group *P* value P(Perm)	Time point *P* value P(Perm)	Group *P* value P(MC)	Time point *P* value P(MC)	MIND spp. number
>10% per Time point^*b*^					
CHG-TrUn0 *versus* CHG-TrUn1,2,3	0.4038	0.3085	0.3428	0.3656	173
CHG-TrUn0 *versus* CHG-TrUn_avg_	0.3207	0.0611	0.3254	0.0674	173
>10% across all Time points^*b*^					
CHG-TrUn0 *versus* CHG-TrUn1,2,3	0.2149	0.0262	0.2223	0.0336	27
CHG-TrUn0 *versus* CHG-TrUn_avg_	0.3218	0.1263	0.3218	0.1450	27

a PERMANOVA results from relative abundance matrices were square root transformed with generated Bray Curtis similarity matrices. PERMANOVAs were implemented using a mixed-model design, unrestricted permutation of raw data, 9,999 permutations, and a type III partial sum of squares. The PERMANOVA implemented a three-factor fixed design consisting of “Group” (CHG-Treated *versus* CHG-Untreated), “Time point” (T**_0_**, T**_1_**, T**_2_**, and T**_3_**), and “Subject” (paired left (CHG-Tr) and right (CHG-Un) forearms; N = 9 Pts).

b Main MIND network refers to species connected to the largest numbers of nodes and were created from following inputs: (i) species were present in at least 10% sample per time point (1 of 9 HVs) per group CHG-Treated (CHG-Tr: 229, 231, 187, and 273 spp., respectively) and CHG-Untreated (CHG-Un: 195, 250, 158, and 203 spp., respectively) and (ii) where species were present in at least 10% of samples (4 of 35 samples per either group) across all time points combined (n = 52). Comparisons included pretreatment (CHG-TrUn0) compared to post treatment (CHG-TrUn1,2,3) and the average RA of post treatment (CHG-TrUn_avg_). P(perm) is permutation-based *P* value, P(MC) is Monte-Carlo *P* value.

In the second (ii) comparison (Fig. S2b), CHG-Tr0 and CHG-Un0 MINDs were very similar, but different from the CHG-Tr1 and CHG-Un1 MINDs. The results suggest that sampling was performed consistently between CHG-treated and CHG-untreated arm and that a similar change has occurred in both arms from T0 to T1, despite of CHG-untreated arm not being covered by AquaGuard protector covers compared left CHG treated arm. MINDs obtained for CHG-Tr2, CHG-Un2, CHG-Tr3, and CHG-Un3, could indicate sampling inconsistency, since CHG-Tr3 MIND is similar to CHG-Un2 MIND, which paradoxically had missing data for one HV. PERMANOVA performed on 27 of the 52 species, representing the main network nodes, detected a significant skin microbiome change over time in the comparison CHG-TrUn0 *versus* CHG-TrUn1, 2, 3 (P(perm)=0.0262; P(MC)= 0.0336), which did not distinguish the CHG-treated from the CHG-untreated arm (Table 4). Taking into consideration the results of both comparisons (i and ii), small changes in species listings along with sampling inconsistencies significantly altered network appearance and produced inconclusive PERMANOVA results. This confirmed the absence of a large and consistent CHG effect size for the time series studied.

### LEfSe analysis for the detection of distinctive species.

**FIG 2:**
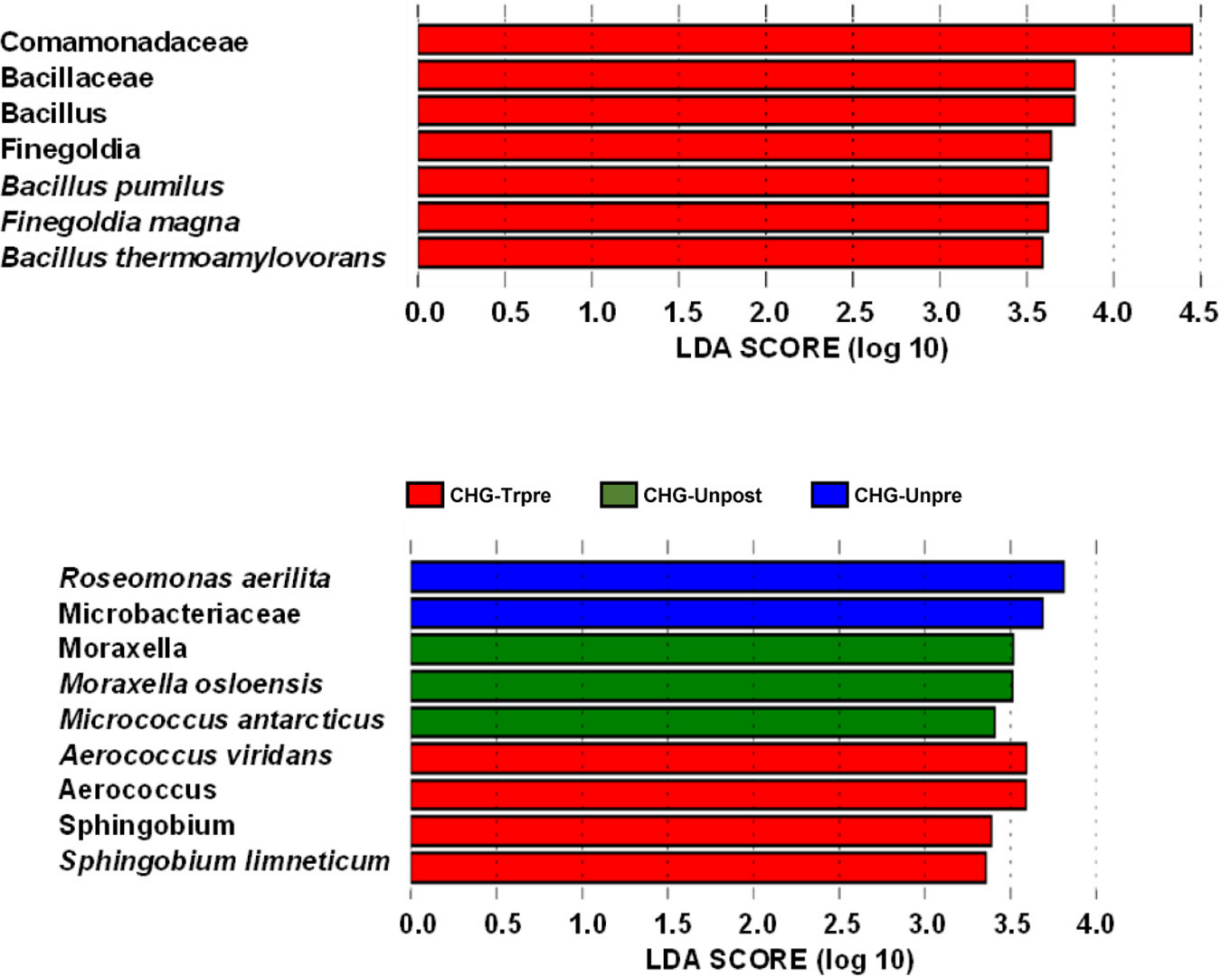
LEfSe analysis CHG-Treated *versus* CHG-Untreated, all time points (T_0_ *versus* T_1_, T_2_, T_3_), all species identified (n = 950). (a) CHG-Tr_0,1,2,3_ *versus* CHG-Un_0,1,2,3_. (b) CHG-Tr_pre_/CHG-Tr_post_ *versus* CHG-Un_pre_/CHG-Un_post_. LEfSe analysis was performed with an input of all 950 species detected across time points. Group was used as “class” and patient was used as “subject.” Strategy was set to “one-against-all” strategy for multiclass analysis, the factorial Kruskal–Wallis test and pairwise Wilcoxon signed rank tests were set at a Monte-Carlo significance level α = 0.05 to calculate LDA scores. The log LDA score was set at a threshold >0. (a) Comparison in which LEfSe data input consisted of “Group”, i.e., CHG-Treated (CHG-Tr) *versus* CHG-Untreated (CHG-Un) all time points for the input option “Class” and “HV” for the LEfSe input option “Subject.” (b) Data input consisted of “Group”, i.e., CHG-Tr_pre_ (before treatment), CHG-Tr_post_ (after treatment) *versus* CHG-Un_pre_ (before treatment), CHG-Un_post_ (after treatment) and “HV” (healthy volunteers) for the LEfSe input option “Subject”. Histograms of significant distinctive features are shown in (a) for CHG-Treated (red bars) and (b) for CHG-Treated and CHG-Untreated arm (red: CHG-Tr_pre_; blue CHG-Un_pre_; and green: CHG-Un_post_, no feature for CHG-Tr_post_).

LEfSe analysis performed in the comparison CHG-Tr0,1,2,3 *versus* CHG-Un0,1,2,3 based on an input of all species detected across all time points n = 950, showed only seven (<1%) significant distinctive taxa for the treated left arm (Fig. 2). The distinctive species were Finegoldia magna, Bacillus thermoamylovorans, and Bacillus pumilus with an LDA SCORE exceeding 3.5. No distinctive taxon was identified for the untreated right arm. The three species were detected at all time points, corresponding to species presence in least 1.25 sample per time point, on average.

Examination of detection and relative abundance data showed that *F. ma*gna had higher representation, while *B. pumilus* had both higher representation and average relative abundance, post-CHG treatment in the CHG-treated compared to the CHG-untreated arm (Table 5). In addition, for the three species, the average relative abundance in the CHG-treated arm was significantly higher than the CHG-untreated arm, post-CHG treatment (*P* < 0.05, Wilcoxon signed rank test, data not shown).

**TABLE 5:** Analysis of pre- and post-CHG treatment changes for the LEfSe identified species Finegoldia magna, Bacillus thermoamylovorans, and Bacillus pumilus^*a*^

Species	T_0_ number S/9 TS^*b*^	T_1_,T_2_,T_3_ number S/26 TS^*b*^	T_1_,T_2_,T_3_/T_0_ number spl.^*c*^	T_1_,T_2_,T_3_/T_0_ avg RA^*d*^	No. spl. change (CHG-Treated/CHG-Untreated)^*e*^	Avg RA change (ratio)^*f*^
CHG-Treated						
*F. magna*	2	10	1.87	2.35	1.25	0.95
*B. thermoamylovorans*	4	8	0.75	2.19	1.33	39
*B. pumilus*	3	9	1.12	5.03	2	43
CHG-Untreated					
*F. magna*	1	4	1.50	2.46		
*B. thermoamylovorans*	2	3	0.56	0.06		
*B. pumilus*	2	3	0.56	0.12		

a Comparisons were made to determine the magnitude of change in sample representation and relative abundance pre- *versus* post-CHG treatment for the three CHG-Treated/left arm distinctive species identified by LEfSe analysis.

b Detection of species pre- (T**_0_**) and post-CHG (T**_1_**,T**_2_**,T**_3_**) treatment is shown. None of the three species were detected in CHG-Treated arm of HV-210 who had no CHG-Untreated/right arm data and was excluded from paired analysis (*i.e.,* 26 paired samples posttreatment).

c Change in representation posttreatment/pretreatment ratio.

d Change in average (avg) relative abundance posttreatment/pretreatment ratio.

e Difference in representation change CHG-Treated/CHG-Untreated group ratio.

f Difference in relative abundance (RA) change CHG-Treated/CHG-Untreated group ratio.

In the second analysis CHG-Tr-pretreatment/CHG-Tr-posttreatment *versus* CHG-Un-pretreatment/CHG-Un-posttreatment, the three species Roseomonas aerilata, Moraxella osloensis, and Micrococcus antarcticus were distinctive of the CHG-untreated arm, while Aerococcus viridans and *Spynghobium limneticum* were distinctive of the CHG-treated arm. However, the species characterizing the CHG-treated arm were not detected in the CHG-untreated arm across all time points, while the species characterizing the CHG-untreated arm were not detected in CHG-treated arm except for Moraxella osloensis detected in 3 posttreatment samples at two time points but not at T0. The data indicate excessive zero-inflation and most likely LEfSe false positives. Therefore, analysis of CHG-Tr *versus* CHG-Un changes in sample representation and relative abundance pre- *versus* posttreatment were not performed.

## DISCUSSION

There are very few longitudinal studies that have determined chlorhexidine treatment effects on skin microbiome using 16S-rRNA gene next generation sequencing. In this pilot study, we identified 950 species level taxa having sampled the dorsal surface area of the left forearm site (treated and covered with cotton dressing) and right forearm site (untreated and exposed) (Table 6). We also detected 273 genera, including species level taxa found predominant in previous skin microbiome studies (15, 16).

**TABLE 6:** Previous skin microbiome of arm studies *ver*sus present chlorohexidine pilot study

Article^*a*^	OTU identification^*b*^	Skin sites^*c*^	Sequencing platform^*d*^	PMID^*e*^
Gao et al., 2007	Phyla: 10 Genera: 119 Species: 247	Volar forearm	Broad-range small subunit 16S rRNA genes PCR-based sequencing of randomly selected clones	17293459
Grice et al., 2008	97% identity: 113 OTUs 99% identity: 261 OTUs Swabs: 1000 bacteria/cm2 Scrapes: 50000 bacteria/cm2 Punch biopsy specimen: 1000000 bacteria/cm2	Swab: 68 Scrape: 55 Punch: 76 Overlapping: 36 Inner elbow	16S rRNA gene survey	18502944
Costello et al., 2009	High diversity locations harbored more phylotypes and more phylogenetic diversity than the gut or oral cavity. High diversity skin sites included the forearm, palm, index finger, back of the knee, sole of the foot.	18 skin locations	Variable region (V2) 16S rRNA gene sequencing	19892944
Capone et al., 2011	Top 30 genera chosen	Swabs from arm, buttock, and forehead of infants	16S rRNA gene PCR-based sequencing	21697884
HMP et al., 2012	Significant associations- Phyla: 5 Genera: 27 Species: 5	Two retroauricular creases, two antecubital fossae, one anterior nares	Illumina 16S rRNA gene pyrosequencing/shotgun sequencing	22699609
Ross et al., 2017	Phyla: 41 phyla Genera: 595 Species: 225	Swabs from 10 skin sites	Illumina V3-V4 16S rRNA gene sequencing	28761935
SanMiguel et al., 2018	Families: top 16 Genera: top 15	Forearm and back	Illumina V1-V3 rRNA gene sequencing	29753031
Kates et al., 2019	Top 5 genera from each group/site and 15 differentially abundant microbiota	Swabs from axillae and antecubital fossae from pediatric and adult patients	Illumina V4 16S rRNA gene sequencing	30879799
This Chlorhexidine Pilot Study - Mougeot et al., 2021	Phyla: 16 Total genera taxa: 373 Total species taxa: 950 Genera nonzeros both arms: 287 Species nonzeros both arms: 643	Left and right forearms	Illumina V1-V3 16S rRNA gene sequencing	NA

a Previous skin microbiome studies of skin sites including the arm using 16S rRNA gene sequencing are listed.

b Operational taxonomic unit (OTU) identification.

c Skin sites sampled.

d Sequencing platform used.

e PMID is PubMed Identification number from the NIH National Library of Medicine. HMP stands for Human Microbiome Project consortium. NA is nonapplicable.

In our study, we found no major effect of CHG treatment on the overall bacterial community structure in terms of relative abundance differences, *alpha*-diversity, or *beta*-diversity, which could be due to the fact that our experimental design consisted of a single daily sampling event over a period of 72h, following one single CHG treatment at baseline.

The first possible explanation is that chlorhexidine does not completely kill the bacteria which allows regrowth of bacteria can occur with only one treatment of chlorhexidine on skin. Indeed, Basher et al. showed some regrowth at 24h after a single chlorhexidine treatment; the authors only showed suppression with daily treatments (13). Safdar et al. showed that using chlorhexidine impregnated dressing with persistent chlorhexidine was more effective in preventing central line infections than one treatment with chlorhexidine (17). Kates et al. was able to identify 15 differentially abundant operational taxonomic units from the control group of antecubital fossae within 23h of a CHG bath (18). Climo et al. showed that daily chlorhexidine baths were required to lower nosocomial infection rates (19). Chlorhexidine remains effective in preventing surgical site infections (20, 21). Our data do not imply that it is ineffective.

The second possibility is that we were measuring live and dead bacteria since dead and live bacteria cannot be distinguished using a metagenomic sequencing approach. Therefore, small CHG treatment associated microbiome profile changes may be overshadowed by a low signal to noise ratio. The cationic and nonvolatile form of chlorhexidine indeed strongly binds to skin and negatively charged bacteria, thereby preventing dead complexed bacteria to be effectively shed from the left arm treated skin that has been covered with dressing and cover protector for showering during the time course of the entire study (22). Furthermore, DNA from dead bacteria may act as a varnish which can intensify the issue, but this possibility is difficult to measure. Indeed, a study by SanMiguel et al. showed that treatment of chlorhexidine on the forearm confounds DNA-based metrics after initial analyses indicated negligible effects on the skin microbiome (23). However, while shedding may be limited, previous studies have shown significant regrowth by 24h after CHG treatment (13, 14).

Higher species identification may be attributed to multiple time points sampling and/or the use of V1-V3 16S rRNA sequencing platform instead of V3-V4. Previous studies have suggested that phylotype richness using V1-V3 was superior to V3-V4 (24, 25). Indeed, skin microbiome sampling generally yields low biomass (26). Furthermore, there are still ongoing investigations in the field to determine which methods would be best to sample and sequence the skin microbiome, considering that skin consists of sebaceous (oily), dry, intermittently moist, and moist microenvironments. Popular methods include swabbing, tape-stripping, and scraping, all producing relatively low biomass samples (27, 28). A study comparing swabbing and tape-stripping yielded an average Yue and Clayton theta index (fYC) of 0.67 and a Pearson’s correlation coefficient (*r*= 0.86), at the genus level (27). Another study found that swabbing was more consistent than scrapping, generating better correlation coefficients (Pearson’s *r* and Spearman’s *rho*) at all taxonomic levels (28). In general, the skin microbiome is subjected to exposure to external environmental factors to a greater extent than oral or gut microbiome. For this reason, higher variability and zero-inflation of the microbiome data may be observed, even when bacterial DNA samples are analyzed by most performant DNA extraction methods and next generation metagenomic sequencing approaches.

Previous studies have shown that effective antimicrobial concentrations of CHG, measured by *in vitro* bacterial cultures, remain on skin at least 24h after application (even after a water rinse), thereby reducing lower central venous catheter-associated bloodstream infection (29, 30). Thus, there was a reasonable assumption to anticipate a detectable CHG effect on skin microbiome after 24h in our study. While such effects would not be to the extent of those incurred by impregnated CHG dressing (17), they might have corresponded to limited bacterial regrowth as described by Basher et al. (e.g., including by CHG resistant species) or corresponded to the suppression of the more susceptible bacterial species (13).

That said, we hypothesized that CHG resistant bacteria would become prominent after one treatment of CHG, and no study had investigated the effects of CHG directly on the forearm microbiome using a metagenomic sequencing approach. In our analysis, using relative abundance data from all the species taxa detected (n = 950), the PERMANOVA comparison CHG-TrUn0 *versus* CHG-TrUn1,2,3 (Table 3) distinguished CHG-treated from CHG-untreated arm marginally, without a significant change over time, while the same comparison using network-associated species (n = 27) present in at least 10% of samples across all time points (Table 4) identified a change over time, but no difference between CHG-treated and CHG-untreated arm overall. A significant and measurable CHG effect would likely have shown a difference between the CHG-treated and CHG-untreated arm and between time points.

Interestingly, although LEfSe results will need future confirmation, they suggested the increased presence of the clinically/industrially relevant and possibly opportunistic species: Bacillus pumilus, Bacillus thermoamylovorans, and Finegoldia magna, the only species of the 950 taxa detected found significant by LEfSe (Figure 2a). We determined that there was a higher representation of *B. pumilus* and *B. thermoamylovorans* post-CHG treatment (100% [CHG-Tr/Un = 2] and 33% [CHG-Tr/Un = 1.33], respectively) and on average an ∼2000% [CHG-Tr/Un∼40] increase in relative abundance, in CHG-treated compared to CHG-untreated arm (Table 5). Previous studies have demonstrated CHG resistance among other Bacillus spp., therefore *B. pumilus* and *B. thermoamylovorans* may have some CHG resistance (31). *F. magna* post-CHG sample representation increased by about 25% [CHG-Tr/Un = 1.25] more in the CHG-treated arm than in the CHG-untreated arm. None of these bacterial species are common clinical pathogens, but they have been occasionally associated with infections. *B. pumilus* has been occasionally associated with infections producing cutaneous anthrax-like lesions, catheter infections, and severe sepsis in neonatal infants (32–35). *B. pumilus* was previously shown to be extremely resistant to oxidative stress, which defines CHG antimicrobial activity (reactive oxygen species [ROS] elevation and lipid peroxidation) (36, 37). It is, therefore, conceivable that in our study *B. pumilus* abundance/presence was increased by negative selection. *B. thermoamylovorans* produces enzymes linked to food spoilage (38). It is resistant to ultrahigh temperature treatment of milk and is therefore considered a threat to the dairy industry (38). *F. magna* is a well-known opportunistic pathogen able to induce inflammation by causing neutrophils to release ROS (39). *F. magna* has been associated with bone and joint infections in immunocompromised patients (39, 40). Notably, Burnham et al. found that *F. magna* and *Bacillus* spp. were among the common skin bacteria in their culture-based study (14). Cassir et al. found *Bacillus* spp. to be more common in the CHG treated group (12).

The most likely source of these bacteria after CHG treatment and under sterile dressings is that CHG did not kill the bacteria with one treatment. *Bacillus* spp. have been associated with CHG resistance (31) so their presence after CHG treatment is likely due to some resistance. Another possible source is that the bacteria are sequestered in hair follicles and then replenish the normal skin microbes. In the same way that the appendix may act as a reservoir to replenish the colonic microbes (41), hair follicles might perform the same function for the skin. Finally, bacterial species might enter under the edges of the dressing.

### Limitations.

In the present study, excessive adhesion of chlorhexidine to skin may have prevented the detection of strong antimicrobial effects on the skin microbiome using a metagenomic approach.

In addition, the significance of our findings remains elusive, as many species are not systematically detected on the CHG-treated and CHG-untreated arm at every time point for the same individual. The sampling sites on consecutive days overlapped so previous skin sampling might have affected measurements.

Understanding the microbiome under sterile dressings is crucial to preventing surgical site infections and intravenous line infection. The CHG-treated arm was covered with cotton gauze and AquaGuard while the CHG-untreated arm remained uncovered. There is a possibility that changing the AquaGuard may have led to contamination of the CHG-treated area. In addition, covering the arm skin for 3 days could also affect the microbiome independently. Moreover, due to the low biomass inherent to skin microbiome, a variable number of PCR cycles were required to prepare the sequencing libraries. This would potentially increase the likelihood of detecting species from contamination. We plan to revise, extend, and broaden these studies with a larger sample size.

Alternative targeted approaches to analyze specific bacterial interaction networks using omics technologies (i.e., host/bacteria RNA, DNA, protein, metabolites), along with *in vitro* culture assays, may also be devised. Such methods, however, will require significant expansion of the databases containing records of curated bacterial interactions.

### Conclusions.

In our pilot study, these findings suggest that the skin microbiome was not significantly different in the 24, 48, or 72h after one treatment with chlorhexidine compared to control specimens, but our assays may have measured nonviable bacteria. Previous culture-based studies (13, 14) have also shown no change at 24h. Three bacterial species are noted in the specimens after CHG treatment; their significance will need to be clarified regarding their resistance to CHG treatment. Targeted approaches may help better characterize bacterial interaction networks and associated changes regarding the presence and activity of opportunistic pathogens following chlorhexidine treatment.

## MATERIALS AND METHODS

### Subject recruitment.

Healthy Volunteers were recruited at Carolinas Medical Center–Atrium Health. Skin swabs were obtained from 10 healthy volunteers (6 males; 4 females) at 24h intervals over 72h to analyze microbiome changes following chlorhexidine treatment of a dorsal surface area of the left forearm (CHG-treated; CHG-Tr) compared to the right forearm (CHG-untreated; CHG-Un) equivalent (paired) dorsal surface area. The area wiped with chlorhexidine impregnated cloth (Sage 2% Chlorhexidine Gluconate [CHG] Cloths, Sage Products, Cary, IL) was covered with 4“x4” sterile Curity cotton dressing (Covidien, Dublin Ireland), while the right arm paired untreated area remained uncovered. All volunteers provided written informed consent, and the protocol was approved by the Atrium Health institutional review board (IRB protocol number 06-17-30E). A questionnaire was used to collect clinical information, including age, gender, race, weight, height, body-mass index, and recent antibiotic use. Volunteers were included in the study if they were age 18 or older and able to keep the left forearm treated area dry using protector covers and return for daily sampling over a 72h period. Volunteers were excluded from the study if they had an active infection in the last 2 weeks on either arm, had an absence of an arm or major defects from past trauma to the arms, had an allergy to chlorhexidine, paper tape, or cotton dressing, were unable to keep AquaGuard covered arm dry, had a hospitalization within the last 2 months, or practiced use of antibacterial soap on the arms in the last 2 weeks.

### Skin treatment with chlorhexidine, sample collection, and processing.

**(i) Skin Treatment with Chlorhexidine Wipe.** Shortly after the day 0 sample was collected, the dorsal surface of the left forearm was prepped with a wipe Sage 2% Chlorhexidine Gluconate (CHG) Cloth (Sage Products, Cary, IL) for 3 strokes followed by 3 min of drying time over a 10x8cm area on the left arm. The corners of the treated area were marked. Chlorhexidine was not applied to any open sores. The left arm treated area was covered with several 4“x4” sterile Curity cotton dressing (Covidien, Dublin Ireland) to cover the 10x8cm dorsal forearm area adequately. The subjects were also given four disposable 7“x7” AquaGuard protector covers (Covalon Technologies, Ltd., Mississauga, ON, Canada) and paper tape to cover the left arm during showering. The right arm received no chlorhexidine wipe treatment, was not covered with sterile cotton dressing, and was not protected with covers during showering. The sampling sites on consecutive days sometimes overlapped.

**(ii) Specimen Collection**. Swab samples for skin microbiome were taken from the dorsal surface of both forearms. Initial samples were collected shortly prior to chlorhexidine treatment administered on the same surface, since our assumption was that skin follicles represented the most consistent reactive source of the skin microbiome (Time T**_0_**). Similar to a procedure described by Horton et al. (42), two (pooled) specimens per left/right arm site were obtained by swabbing for 45s with a sterile cotton swab soaked in saline with Tween (ST) solution (0.15 M NaCl with 0.1% Tween 20). The head of each swab was aseptically cut from the handle, placed in a microcentrifuge tube containing 100 μL of ST solution, and centrifuged (8000 RPMs; 5 min). The swab was removed, the pellet and supernatant were separated, and the pellet was stored at −80°C. The subjects returned at 24, 48 and 72h (T**_1_**, T**_2_**, and T**_3_**; +/-4 h each day) for two swab specimens per site on the dorsal surface of both forearms. Samples were labeled CHG-Tr0 to CHG-Tr3 and CHG-Un0 to CHG-Un3, associated with a de-identified subject code 201 to 210.

**(iii) Sample Processing and Sequencing**. Bacterial DNA was extracted from frozen pellets using the ZymoBIOMICS -96 MagBead DNA kit (Zymo Research, Irvine, CA). Quick-16S Primer Set V1-V3 (Zymo Research, Irvine, CA) was used to prepare the sequencing library using real-time PCR to control cycle number and limit PCR chimera formation (Table S1). Final PCR products were quantified, pooled and the library was cleaned with Select-a-Size DNA Clean & Concentrator (Zymo Research, Irvine, CA). Final quantification was performed with TapeStation (Agilent Technologies, Santa Clara, CA) and Qubit (Thermo Fisher Scientific, Waltham, MA). Along with negative controls for both DNA extraction and library preparation processes, the ZymoBIOMICS Microbial Community Standard (Zymo Research, Irvine, CA) was used as a positive control for each DNA extraction and the ZymoBIOMICS Microbial Community DNA Standard (Zymo Research, Irvine, CA) was used as a positive control for each targeted library preparation. The final library was sequenced on Illumina MiSeq with a v3 reagent kit (600 cycles). The sequencing was performed with >10% PhiX spike-in.

### Bioinformatics analysis.

Unique amplicon sequences were inferred from raw reads and chimeric sequences were removed using the Dada2 pipeline (Table S2) (43). Taxonomy assignment and associated relative abundances were determined using Uclust from Qiime**_v.1.9.1_** (44). Taxonomy was assigned with the Zymo Research Database, a 16S database that is internally designed and curated, as reference. PERMANOVA analyses were performed PRIMER**_v7_** program (PRIMER-E Ltd., Ivybridge, UK) to compare T**_0_** CHG-TrUn0 data to CHG-TrUn data from posttreatment time points T**_1_**, T**_2_**, and T**_3,_** alone or in combinations. An additional comparison with CHG-TrUn0 was made in which T**_1_**, T**_2_**, and T**_3_** posttreatment relative abundance data were averaged to represent a “single” posttreatment time point.

Relative abundance matrices were square root transformed and Bray Curtis similarity matrices were generated. PERMANOVAs were implemented using a mixed-model design, unrestricted permutation of raw data, 9,999 permutations, and a type III partial sum of squares. The PERMANOVA implemented a three-factor fixed design consisting of “Group” (Treatment *versus* No Treatment), “Time point (T0, T1, and/or T2, and/or T3), and “Subject” (paired left and right forearms). P(perm) and P-Monte-Carlo (P[MC]) significance levels were set at α = 0.05.

Wilcoxon signed rank (WSR) test was used to compare relative abundances of species between left and right arm for each time point for available paired data (https://www.socscistatistics.com, significance level α = 0.05).

Linear discriminant analysis (LDA) effect size (LEfSe) was performed as previously described for the comparisons CHG-treated *versus* CHG-untreated all time points and CHG-Tr-pretreatment, CHG-Tr-posttreatment *versus* CHG-Un-pretreatment, CHG-Un-posttreatment (45, 46).

In the LEfSe online program Galaxy**_v1.0_**, LEfSe data input consisted of ‘Group’ defined by CHG-Tr1, CHG-Tr2, CHG-Tr3, CHG-Tr4 and CHG-Un1, CHG-Un2, CHG-Un3, CHG-Un4 for the input option “Class” and “HV” for the input option “Subject.” Alternatively, “Group” was defined by CHG-Tr-pretreatment, CHG-Tr-posttreatment, CHG-Un-pretreatment, and CHG-Un-posttreatment.

Using the “one-against-all” strategy for multiclass analysis (46), the factorial Kruskal–Wallis test and pairwise Wilcoxon signed rank tests were set at a PMC significance level α = 0.05 to calculate LDA scores. The log LDA score was set at a threshold >0 and used to generate a cladogram representing the hierarchy of all significant biomarkers and a histogram of the top biomarkers, plotted at the species level for select comparisons found significant *via* PERMANOVA.

Bacterial network analysis was carried out using the microbial interaction network database online tool (MIND_v1.01_; http://www.microbialnet.org/mind.html), integrating concepts of multidimensionality of microbial interactions (47). MIND, which contains records of 523,913 interactions for a total of 5,532 microbes (as of March 18, 2021), uses a list of species as input and generates an interaction network based on known interactions that have been identified in different human body sites. Species identified as part of a theoretical interaction network consistently present across all time points, were used for PERMANOVA for the above-mentioned comparison designs.

### Data availability.

Raw FastQ files related to this project were submitted to NCBI Sequence Read Archive (https://www.ncbi.nlm.nih.gov/bioproject/PRJNA810339). Additionally, raw data are available from the corresponding author.
