# Supplementary material for: Cutaneous Microbiome Profiles Following Chlorhexidine Treatment in a 72-Hour Daily Follow-Up Paired Design: a Pilot Study
Source: Microbiol Spectr. 2022 Jun 21;10(3):e01753-21. doi: 10.1128/spectrum.01753-21 (PMC9248901; doi:10.1128/spectrum.01753-21)
Supplement: SUPPLEMENTAL FILE 1 — Supplemental material. Download spectrum.01753-21-s001.pdf, PDF file, 0.9 MB [file spectrum.01753-21-s001.pdf]

Supplemental Table 1. Amplification results for skin microbiome 16S rRNA gene data

Sample ID (CHG-Treated)	Cycle	Sample ID (CHG-Untreated)	Cycle
201.L0	26	201.R0	34
201.L1	34	201.R1	38
201.L2	38	201.R2	38
201.L3	38	201.R3	34
202.L0	38	202.R0	38
202.L1	>38	202.R1	34
202.L2	34	202.R2	34
202.L3	>38	202.R3	38
203.L0	30	203.R0	30
203.L1	30	203.R1	30
203.L2	30	203.R2	26
203.L3	30	203.R3	26
204.L0	30	204.R0	26
204.L1	26	204.R1	26
204.L2	30	204.R2	30
204.L3	30	204.R3	30
205.L0	26	205.R0	26
205.L1	26	205.R1	26
205.L2	26	205.R2	26
205.L3	26	205.R3	26
206.L0	34	206.R0	34
206.L1	34	206.R1	30
206.L2	34	206.R2	30
206.L3	30	206.R3	30
207.L0	30	207.R0	34
207.L1	34	207.R1	30
207.L2	34	207.R2	30
207.L3	34	207.R3	30
208.L0	34	208.R0	30
208.L1	34	208.R1	30
208.L2	34	208.R2	30
208.L3	34	208.R3	34
209.L0	34	209.R0	34
209.L1	34	209.R1	34
209.L2	34	209.R2	30
209.L3	30	209.R3	42
210.L0	30	210.R0	34
210.L1	30	210.R1	34
210.L2	30	210.R2	34
210.L3	30	210.R3	30
Positive Control	18		
Negative Control	38		

Bacterial DNA was extracted from frozen pellets using the ZymoBIOMICS ®-96 MagBead DNA Kit (Zymo Research, Irvine, CA). Quick-16S™ Primer Set V1-V3 (Zymo Research, Irvine, CA) was used to prepare the sequencing library using real-time PCR to control cycle number and limit PCR chimera formation. Samples were collected at various cycles after sufficient amplification was reached, or at 42 cycles if no amplification occurred.

Supplemental Table 2. Summary of sequence read data processing

Sample[a]	Raw seqs (R1+R2)[b]	Trimmed seqs (R1+R2)[c]	dada2 inferred[d]	Chimera seqs[e]	Chimera free seqs[f]	Unique seqs[g]	Seqs (after_size_filtration)[h]	Final unique seqs[i]
**201.L0**	61628	58578	26725	25	26700	75	26661	69
**201.L1**	69868	66736	33092	980	32112	37	32112	37
**201.L2**	68984	65078	32387	15	32372	9	32372	9
**201.L3**	123316	116886	57309	529	56780	32	56780	32
**201.R0**	66354	63052	30770	13	30757	56	30757	56
**201.R1**	120160	111046	53866	354	53512	25	53512	25
**201.R2**	95588	91240	45010	411	44599	21	44599	21
**201.R3**	76062	72136	35785	714	35071	36	35071	36
**202.L0**	81472	76154	37392	259	37133	19	37133	19
**202.L1**	52068	49702	24788	0	24788	5	24788	5
**202.L2**	99382	93588	44797	264	44533	68	44532	67
**202.L3**	52904	49912	24923	0	24923	5	24923	5
**202.R0**	63940	58524	26688	33	26655	11	26655	11
**202.R1**	93688	89206	44393	2657	41736	22	41736	22
**202.R2**	92216	87978	43095	690	42405	59	42398	58
**202.R3**	99356	94798	46975	49	46926	20	46916	19
**203.L0**	53130	49156	23016	39	22977	40	22975	38
**203.L1**	61656	57678	27067	109	26958	66	26934	61
**203.L2**	31070	28886	12732	17	12715	43	12712	42
**203.L3**	32298	30076	13994	223	13771	41	13771	41
**203.R0**	73918	68986	33194	1388	31806	55	31784	51
**203.R1**	94296	87600	41367	50	41317	75	41277	69
**203.R2**	40128	37626	18263	5	18258	10	18258	10
**203.R3**	30116	28222	13310	45	13265	46	13247	43
**204.L0**	74668	70010	34381	2944	31437	18	31413	13
**204.L1**	32694	30786	15040	0	15040	4	15031	3
**204.L2**	59568	56092	27370	2	27368	16	27352	13
**204.L3**	63864	60070	28082	3	28079	30	28073	28
**204.R0**	28154	26466	12572	0	12572	9	12572	9
**204.R1**	28038	26248	12846	0	12846	8	12836	7
**204.R2**	55312	52222	25514	0	25514	9	25514	9
**204.R3**	70284	66276	31554	20	31534	41	31511	38
**205.L0**	44928	42190	20978	0	20978	3	20978	3
**205.L1**	22516	21222	10399	0	10399	5	10383	3
**205.L2**	21448	20156	10057	0	10057	1	10057	1
**205.L3**	54990	51696	25716	0	25716	2	25716	2
**205.R0**	34874	32830	16336	0	16336	1	16336	1
**205.R1**	28244	26572	13233	0	13233	1	13233	1
**205.R2**	26854	25326	12617	0	12617	2	12617	2
**205.R3**	31678	29894	14856	0	14856	1	14856	1
**206.L0**	57408	53696	26116	159	25957	43	25954	42
**206.L1**	68864	64238	30145	217	29928	54	29927	53

**206.L2**	65516	61142	28525	134	28391	53	28391	53
**206.L3**	50296	46854	20776	151	20625	77	20561	65
**206.R0**	42536	39814	17856	0	17856	51	17842	48
**206.R1**	32116	29548	13512	2	13510	35	13494	32
**206.R2**	44048	41380	20095	0	20095	26	20095	26
**206.R3**	34550	32496	15252	48	15204	53	15194	51
**207.L0**	32500	30332	12968	3	12965	69	12961	66
**207.L1**	62444	58406	27749	413	27336	71	27327	70
**207.L2**	41768	39016	18336	37	18299	35	18299	35
**207.L3**	65996	61704	29780	1311	28469	80	28456	78
**207.R0**	47912	44636	21433	354	21079	49	21079	49
**207.R1**	58664	54824	26268	14	26254	59	26199	51
**207.R2**	44270	41456	19217	290	18927	84	18893	80
**207.R3**	61178	57348	26033	298	25735	88	25723	85
**208.L0**	63134	59388	28754	1524	27230	49	27227	48
**208.L1**	39334	36738	17543	14	17529	40	17520	39
**208.L2**	34604	32204	15264	41	15223	34	15220	33
**208.L3**	57838	53594	22864	231	22633	89	22626	87
**208.R0**	27578	25982	12104	16	12088	33	12080	30
**208.R1**	70244	65394	27114	201	26913	167	26739	136
**208.R2**	36162	34082	16046	154	15892	40	15873	38
**208.R3**	59482	55934	27499	775	26724	45	26724	45
**209.L0**	66554	62846	30065	326	29739	49	29738	48
**209.L1**	76740	72338	35617	35	35582	71	35581	70
**209.L2**	39054	36678	17974	3	17971	25	17969	24
**209.L3**	39186	36706	15686	75	15611	93	15594	88
**209.R0**	56088	52688	25273	4	25269	40	25267	39
**209.R1**	68962	64728	30725	74	30651	52	30644	50
**209.R2**	53762	49858	24036	93	23943	29	23928	27
**209.R3**	34872	28734	12108	1835	10273	24	10228	15
**210.L0**	26620	24942	11334	21	11313	38	11310	36
**210.L1**	32854	30742	14036	806	13230	77	13177	67
**210.L2**	33700	31406	14545	36	14509	38	14491	36
**210.L3**	38012	35478	15305	357	14948	62	14934	54
**210.R0**	73404	68706	32522	1717	30805	42	30805	42
**210.R1**	77972	70924	29577	2173	27404	78	27334	62
**210.R2**	7030	26	0	0	0	0	0	0
**210.R3**	47744	44564	19374	564	18810	55	18802	53

[a]Sample ID; L (CHG-Treated), R (CHG-Untreated); paired samples of 10 (6 male, 4 female) healthy volunteers

[b]The number of raw sequences for each sample (average of 54,783)

[c]The number of sequences after quality filtering

[d]The number of sequences after DADA2 quality control filtering

[e]The number of chimeric sequences identified in the DADA2 inferred sequences

[f]The number of chimera-free sequences identified in the DADA2 inferred sequences

[g]The number of unique sequences in chimera-free sequences

[h]The number of chimera-free sequences that have undergone further amplicon size filtration (data used for QIIME)

[i]The number of unique sequences identified in size-filtered chimera-free sequences.

Supplemental Figure 1. Bacterial interaction network analysis of all species detected in >10% of samples per each timepoint in either group or across all timepoints in both groups.

a. Species detected in >10% samples per timepoint

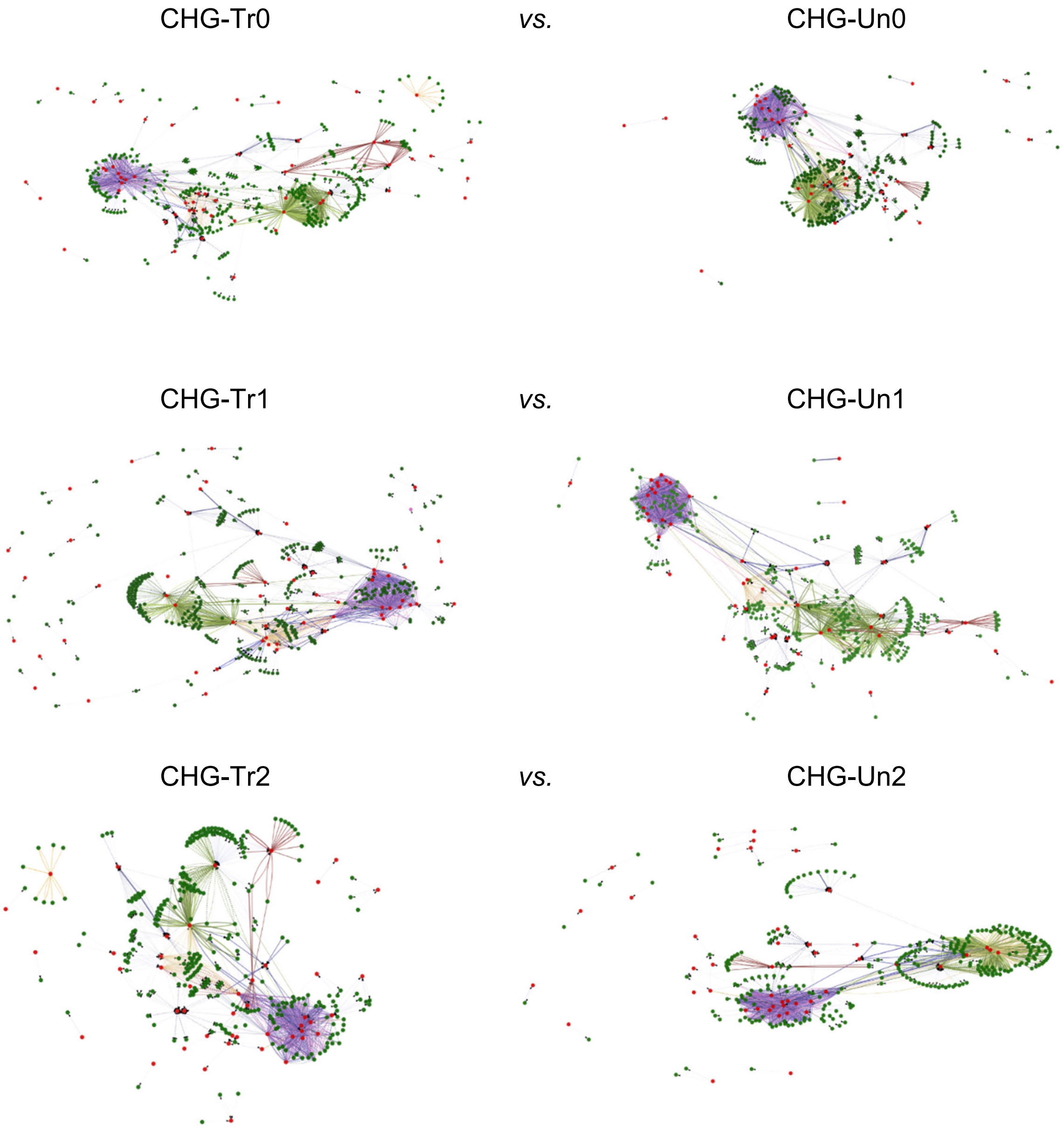

CHG-Tr3 *vs.* CHG-Un3

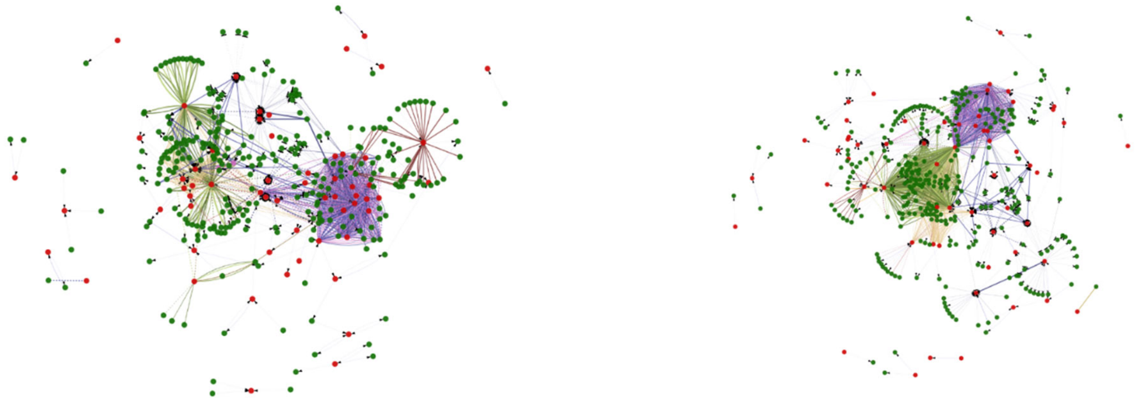

b. Species detected in >10% samples across all timepoints

CHG-Tr0 *vs.* CHG-Un0

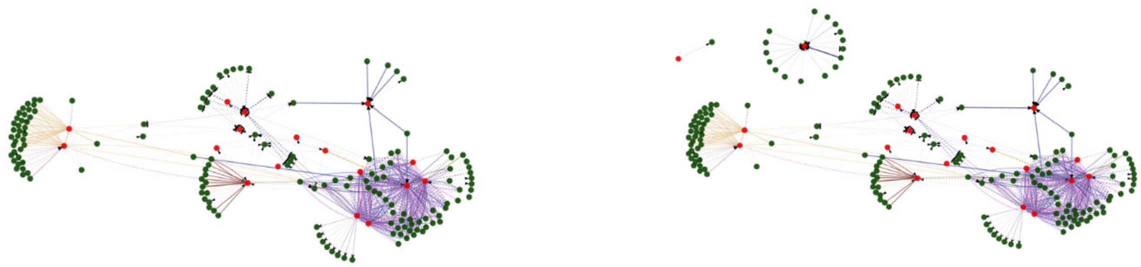

CHG-Tr1 *vs.* CHG-Un1

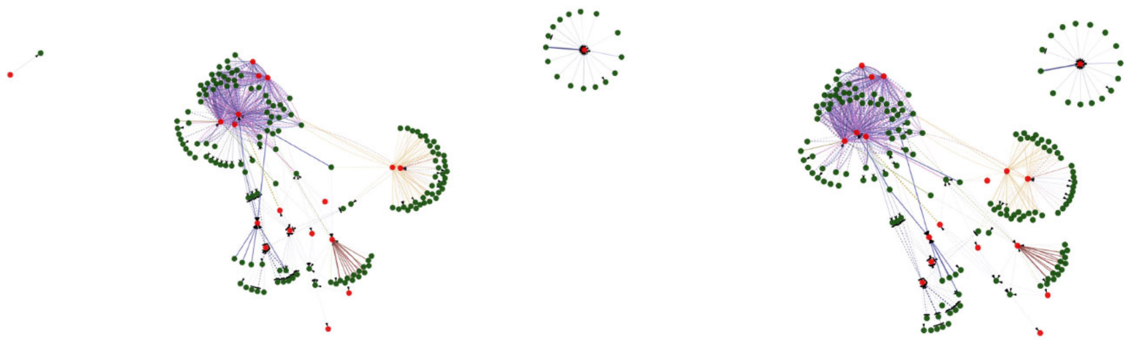

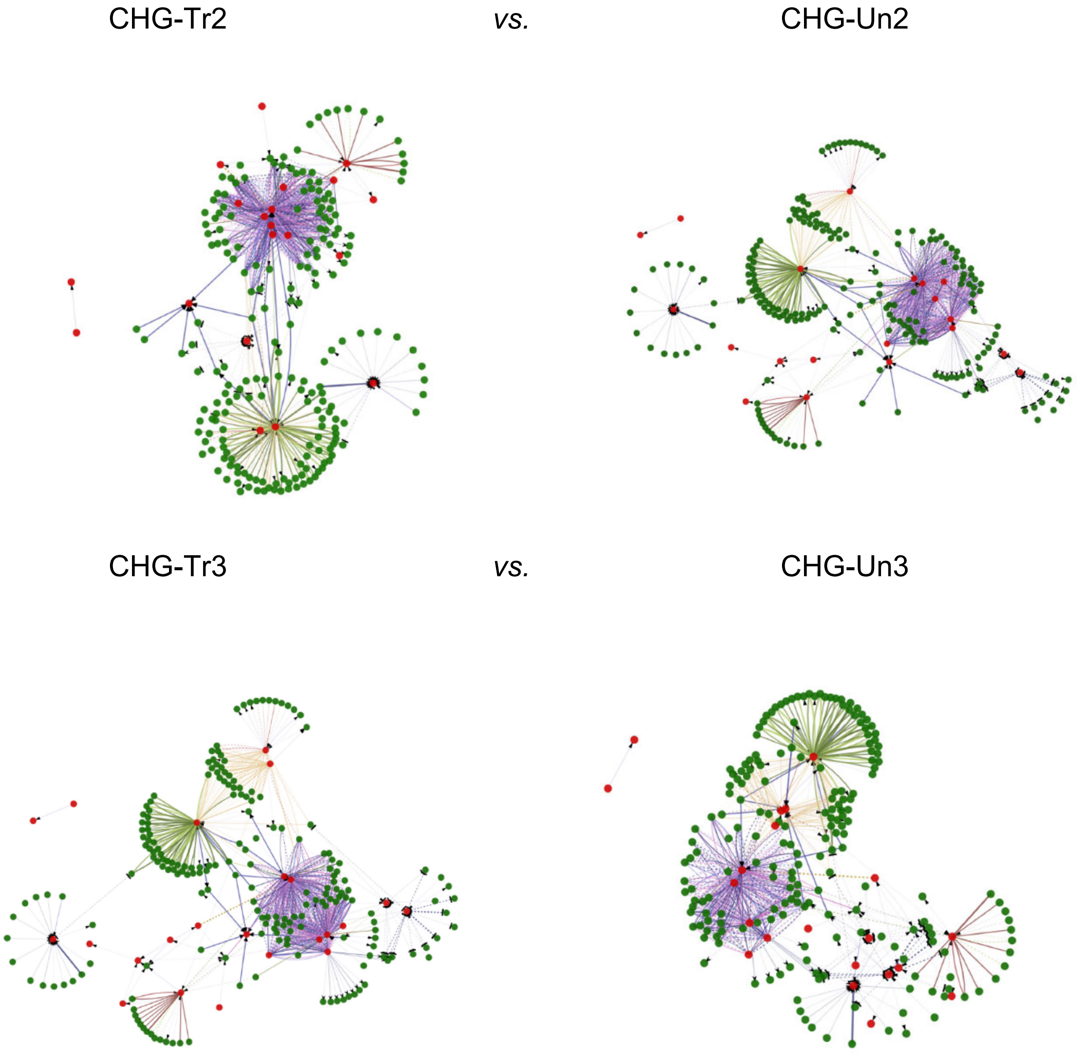

Bacterial interaction networks were generated using $MIND_{v1.01}$ online tool based. (**a**) MIND analysis for the species that are present in at least one out of nine HVs of samples per timepoint T_0, T_1, T_2, and T_3, in either group with CHG-Treated (CHG-Tr) / CHG-Untreated (CHG-Un) input of 229/195, 231/250, 187/158, and 273/203 spp., respectively. (**b**) MIND analysis for the species that are present in at least 4 of 35 samples in both groups with CHG-Tr and CHG-Un input of 52 common species used as input list.
